# A Role for Innexin2 and Innexin3 Proteins from *Spodoptera litura* in Apoptosis

**DOI:** 10.1371/journal.pone.0070456

**Published:** 2013-07-30

**Authors:** Tian Liu, Ming Li, Yan Zhang, Zunyu Pang, Wei Xiao, Yang Yang, Kaijun Luo

**Affiliations:** School of Life Sciences, Key Laboratory for Animal Genetic Diversity and Evolution of High Education in Yunnan Province, Yunnan University, Kunming, Yunnan, P. R. China; Temple University, United States of America

## Abstract

Gap junctions formed by two hemichannels from two neighboring cells are cell-to-cell communication channels; hemichannels are communication channels between intracellular and extracellular environments. Hemichannels are hexameric proteins formed by connexins, pannexins, innexins and vinnexins. Innexin-hemichannels (innexons) exist in the lepidopteran cell surface, but their component innexins and functions have not been reported. Recent studies by others have demonstrated that hemichannels, connexons and pannexons from vertebrates serve as regulators of apoptosis via inactivating the PI3K/Akt signaling pathway. Here, the apoptogenic properties of innexons are demonstrated using two innexin cDNAs, *Spli-inx2* and *Spli-inx3*, which were isolated from hemocytes of lepidopteran *Spodoptera litura*. Alignment analysis revealed that these two genes belong to a conserved innexin family, as they contain the insect signature YYQWV motif at the beginning of the second transmembrane domain. Immunofluorescence showed that two fusion proteins, Inx2-V5 and Inx3-V5, were localized predominantly in the cell membrane, cytoplasm and also nuclei. Ectopic expression in Sf9 cells and over-expression of Inx2 and Inx3 in Spli221 cells promoted apoptosis. In the Spli221 cells, apoptotic cells presented remarkable membrane blebbing. This study also showed that Sf9 and Spli221 cells undergo low level apoptosis under normal culture conditions, but not Hi5 cells. In Hi5 stable cell lines, biotinylation was used to isolate surface proteins and confirm Inx2 and Inx3 localization in the cell membrane and also further data showed that Hi5 cells may activate the PI3K signaling pathway via phosphorylating molecular Akt downstream. This result suggests that innexon-promoted apoptosis may be involving the PI3K/Akt signaling pathway. These findings will facilitate further examinations of the apoptotic regulation by the PI3K/Akt signaling pathway and comparative studies of innexons, connexons, pannexons, and vinnexons.

## Introduction

Gap junctions are docking channels formed by two individual hemichannels from neighboring cells. Hemichannels are hexameric proteins composed of several domains; hemichannel proteins include connexins and pannexins in chordates, innexins in invertebrates [1], [2], [3] and vinnexins in insect-associated ichnoviruses (IVs) [4], [5]. In vertebrates, two hexameric hemichannel proteins, connexon and pannexon, are named based on their constituent domains. Here, we refer to the hexameric innexin proteins as innexons and the hexameric vinnexin protein as vinnexons. Each of the domains in the four types of hemichannels have similar topologies, with four α-helical transmembrane segments (TM), an intracellular loop, two extracellular loops, and an intracellular N- and C-terminal. Two hemichannels from neighboring cells dock together to form a gap junction channel, which is responsible for cell-to-cell communication [6], [7], [8], [9]. Functional homologues of gap junctions have been identified in both vertebrate and invertebrate, but the function of hemichannels has only been identified in vertebrates; in invertebrate, the function is less known.

Opening connexons and pannexons allow the bidirectional passage of ions and small molecules, up to 1–2 kDa, between the extracellular and intracellular space. These affect cellular behaviors through the activation of paracrine signaling pathways [10] via signaling molecules, including ATP [11], [12], [13], [14], [15], ADP, AMP, and Adenosine; the latter three may be degraded by ecto-ATPase [16]. Platelet aggregation depends on the purinergic signaling pathway, which is mediated by ADP. ADP accumulates in the extracellular space to stimulate ADP receptors (G protein-coupled purinergic receptors, namely P2Y1 and P2Y12) [17] in the cell membrane to regulate the downstream PI3K/Akt signaling pathway [18], which activates integrin subunits [19] and trigger aggregation.

Previous studies show the involvement of functional connexons and pannexons in diverse physiological and pathological conditions. Under physiological conditions, connexons and pannexons have a very low opening probability to release physiologically relevant quantities of signaling molecules that activate the paracrine signaling pathway to promote the diffusion of cellular nutrients and/or waste products. Under pathological conditions, the opening of connexons and pannexons may cause the intracellular accumulation of toxic metabolites [20] and ATP depletion [12], [21], which can lead to apoptosis [22], [23], [24] and/or necrotic cell death [25]. Cellular apoptosis involves the inactivation of the PI3K/Akt signaling pathway and activation of caspase cascades [26], [27].

Recently, a functional hemichannel was found to exist in the cell surface of lepidopteran hemocytes [28], and earlier, Gupta [29] documented that gap junctions form during the encapsulation by insect hemocytes. Encapsulation is the primary cellular immune response in insects. During this process, granular cells alter their status from nonadhesive to adhesive in order to encapsulate the foreign target (for instance a wasp egg) and form the first monolayer around wasp’s egg; after encapsulation, the death of wasp’s egg and the granular cells undergo apoptosis [30]. Under normal physiological conditions, insect innexons have a balanced opening probability that maintains normal metabolism. However, under LPS immune challenge, innexons have a reduced opening probability [28]. In addition, under immunosuppression mediated by polydnavirus, granulocytes undergo apoptosis in *S. litura/Microplitis bicoloratus* bracovirus [31] and *Pseudoplusia includens/Microplitis demolitor* bracovirus [32], and vinnexins from *Campoletis sonorensis* ichnovirus still can form hemichannels in the cell membrane to increase the level of cell permeability (personal communication with Dr. Turnbull). The molecular mechanism by which insect innexon regulates the immune response is still less known.

In insects, several reports already showed that paracrine signaling pathway exists in the insect system although the interactions among these proteins are still unclear. Under LPS immune challenge, the capability of hemichannel is decreased in Hi5 cells [28], and the activity of ecto-ATPase is increased in hemocytes of *Manduca sexta* [33]; Meanwhile, the aggregation of hemoctytes increases in *Hyalphora cecropia* [34]. The PI3K/Akt signaling pathway plays a crucial role in the replication of AcMNPV in Sf9 cells [35]. Integrins are responsible for encapsulation during the insect cellular immune response in hemocytes of *M. sexta* [36] and *P. includens* [37].

Here, the apoptogenic properties of innexons were examined using two innexin cDNAs, *Spli-inx2* and *Spli-inx3*, which were isolated from hemocytes of caterpillar *Spodptera litura*. These findings will facilitate the further examination of apoptotic regulation by the PI3K/Akt signaling pathway and comparative studies of innexons, connexons, pannexons, and vinnexons.

## Materials and Methods

### Insect Rearing and Cell Culture

The *S. litura* colony was reared on artificial diet (formulated according to [38] at 27±1°C, RH 60–80%, and under a 12∶12 h photoperiod regimen. Hi5 (BTI-Tn-5B1-4) adherent cells were derived from *Trichoplusia ni* embryos [39], Sf9 (IPLB-Sf21-AE) adherent cells were derived from *Spodoptera frugiperda* pupal ovarian tissue [40], Spli221 (TUAT-Spli221) adherent cells were derived from *S. litura* [41], and the Bm12 (DZNU-Bm-12) cells line was derived from the larval ovaries of *Bombyx mori* [42]. All cell lines were cultured in TNM-FH insect culture medium containing 10% fetal bovine serum (FBS, Hyclone). Cells were maintained and passaged in 25 cm^2^ tissue culture flasks (Corning).

### Total RNA Isolation from Hemocytes and cDNA Synthesis

Fourth instar *S. litura* larvae were used to isolate hemocytes. Total RNA was isolated from 1×10^7^ cells using RNAiso™ Plus (Takara), according to manufacturer’s instructions, including DNase treatment. The concentration and purity of each RNA sample was determined by measuring the OD at A260/A280 using the NanoDrop 2000. Samples with an A260/A280 ratio >2.0) were used to synthesize cDNA using oligo d (T) 18 primers following manufacturer’s instruction (Takara). The cDNA samples were stored at −80°C until use.

### Plasmids and Expression

*Innexin2* (*inx2*) and *innexin3* (*inx3*) were amplified by PCR, using cDNA as a template and the following primers: Inx2_F (5′- ATG TTT GAT GTC TTT GGG TC -3′), Inx2_R (5′-CTA CAC ACT GTC CTT CCC TT-3′), Inx3_F (5′-ATG GCG GTA TTT GGT TTG GT-3′) and Inx3_R (5′-TTA CGT TTC GGT TTC CTT AG-3′). The genes were then directionally cloned into pMD19 and sequenced. To express a SpliINX2 fusion protein with V5 and 6His tags, the following primers were used to make the construct: Spli-inx2_F (5′-GAA TTC ATG TTT GAC GTT TTC GGC T-3′) and Spli-inx2_R (5′-GC GGC CGC ACA CAC TGT CCT T-3′) containing *Eco*R I and *Not* I sites (underline). *Spli-inx2* and *Spli-inx3* genes were sub-cloned into the insect expression plasmid, pIZT/V5-His (Invitrogen) (V5 tag contains 14 amino acid epitope, GKPIPNPLLGLDST, derived from the P and V proteins of the paramyxovirus, SV5), from the T-vector. This insect expression vector uses two promoters: the OpIE2 promoter from *Orgyia pseudotsugata* nucleopolyhedrovirus to express the gene of interest and the OpIE1 promoter to express a Zeocin-green fluorescent protein (GFP) gene fusion protein. The same method was used to make another construct that expressed a SpliINX3 fusion protein, with V5 and 6His tags, using the following primers: Spli-inx3_F (5′-GAA TTC ATG GCG GTA TTT GGT TTG G-3′) and Spli-inx3_R (5′- GC GGC CGC ACG TTT CGG TTT C-3′) containing *Eco*R I and *Not* I sites (underline). The pIZT/V5-His empty vector served as a negative control and was named pIZT.

### Transient Expression of pIZT/Inx2-V5, pIZT/Inx3-V5 and pIZT in Lepidopteran Cells

The constructs were transiently expressed in lepidopteran cells by cationic lipid-mediated transfection. Two hundred thousand cells were seeded in a 12-well culture plate (Corning) 2 h prior to transfection. Cells were transfected using a 4∶1 ratio of X-trem Fugene Transfection Reagent (Roche) (4 µl) and 1 µg DNA per well, per ml, based on the manufacturer’s protocol. Transfection efficiencies ranged from 50 to 75% in different cell lines, as measured by GFP expression.

### Western Blotting

Cells pellets were lysed in cell lysis buffer (50 mM Tris, 150 mM NaCl, 1% Nonidet P-40, pH 7.8), and protein concentration was measured using a Bradford assay. Then, 50 µg of protein was loaded per sample, except where otherwise noted. Polyacrylamide gel electrophoresis and western blotting were performed with 10% gels, and proteins were transferred to PVDF membranes. Recombinant Inx2-V5 and Inx3-V5 fusion proteins were detected with anti-V5 mouse (Invitrogen) (1∶5000) and a goat anti-mouse horseradish peroxidase-conjugated secondary antibody (Beyotime) (1∶2000). Apoptosis was assessed using a cleaved-caspase 3 antibody, which can probe 32 kDa pre-caspase 3 and 17 kDa cleaved caspase 3 at the same time, (Rabbit) (1∶1000) (Anbo) and a goat anti-rabbit horseradish peroxidase-conjugated secondary antibody (Beyotime) (1∶2000). The bands were visualized by chemiluminescence with ECL (Beyotime). The activation of PI3K/Akt signling pathway was checked by using Akt (phosphor-Ser 473) pAb (Rabbit) (Abmart) (1∶1000) and a goat anti-rabbit horseradish peroxidase-conjugated secondary antibody (Beyotime) (1∶5000).

### Immunofluorescence Microscopy

Ninety-six hours post-transfection, 1×10^4^ cells were moved to a 96-well plate. After 24 h, the cells were washed in PBS, and fixed for 15 min in 3.7% formaldehyde. Then, the cells were permeabilized for 10 min with PBT (PBS - 0.2% Triton X-100). Permeable cells were blocked for 1 h in 4% nonfat dry milk in PBS and then incubated 1 h at RT with the anti-V5 antibody (mouse) (Invitrogen) (1∶2000) in PBT. After washing in PBS, the cells were incubated with Alexa fluor 568-conjugated rabbit anti-mouse (1∶2000) (Invitrogen) for 1 h in PBT. Incubation of cells with the primary and secondary antibodies alone served as negative controls. Cells were imaged using an Olympus 71 inverted fluorescence microscope. Apoptogenic cells were detected using an activated caspase 3 antibody, which only identifies 17 kDa cleaved caspase 3, (Rabbit) (1∶1000) (Beyotime) and Daylight 549-conjugated goat anti-rabbit IgG (1∶1000) (Beyotime). A 300 nM final concentration of DAPI was added to each well to stain the cell nucleus prior to imaging.

### Selecting Stably Expressing Inx2-V5, Inx3-V5 Fusion Proteins in Hi5 Cell Lines with Zeocin

The transfection procedure was performed as previously described. The transfected cells were incubated at 27°C; after 96 hrs, the transfected cells were resuspended gently, diluted into TNM-FH 10% FBS medium containing 300 µg/ml Zeocin, and plated in 60-mm dishes at 50% confluency. After 5 days, cell clones stably expressing Inx2-V5, Inx3-V5 and empty vector (pIZT) formed, and the cells were maintained in TNM-FH medium containing 10% FBS and 300 µg/ml Zeocin in 25 cm^2^ flasks. Then, the stable cell lines were verified by western blot and inverted fluorescence microscopy.

### Detection of Cell Surface Proteins in the Cell Membrane of Living Cells

The determination of cell surface proteins Inx2-V5 and Inx3-V5 was performed using the Pierce Cell Surface Protein Isolation Kit (Thermo), according to the manufacturer’s instructions. Four 25-cm^2^ flasks containing cultured cells of interest were washed three times with ice-cold PBS. One vial of Sulfo-NHS-SS-Biotin was dissolved into 48 ml of ice-cold PBS (3 ml was added to each flask), and the flasks were incubated for 30 min at 4°C. The cells were then washed three times with quenching solution. The cells were harvested by centrifugation for 3 min at 500×g at 4°C. Lysis buffer containing a protease inhibitor (200 µg/ml) was added to the cell pellets, and they were incubated for 30 min on ice, with vortexing every 5 min for 5 sec. The cell lysates were then centrifuged for 2 min at 10000×g at 4°C. The resulting supernatants were then moved into 200 µl of NeutrAvidin Agarose slurry and incubated for 1 h at RT. Then, the NeutrAvidin and biotin complexes were washed three times with wash buffer. One hundred and twenty microliters of SDS-PAGE sample buffer containing 50 mM DTT, without bromophenol blue was added to the resin and the samples were placed in a heat block for 5 min at 95°C. The samples were then concentrated for 2 min at 1000×g to collect the eluted protein. After measuring the protein concentration, 0.5% (w/v) bromophenol blue was added prior to western blot analysis, and the remaining protein was stored at −80°C.

## Results and Discussion

### INX2 and INX3 from Lepidopteran Insects Belong to a Conserved Innexin Family Containing a YYQWV Motif at the Beginning of the Second TM Domain

**Figure 1:**
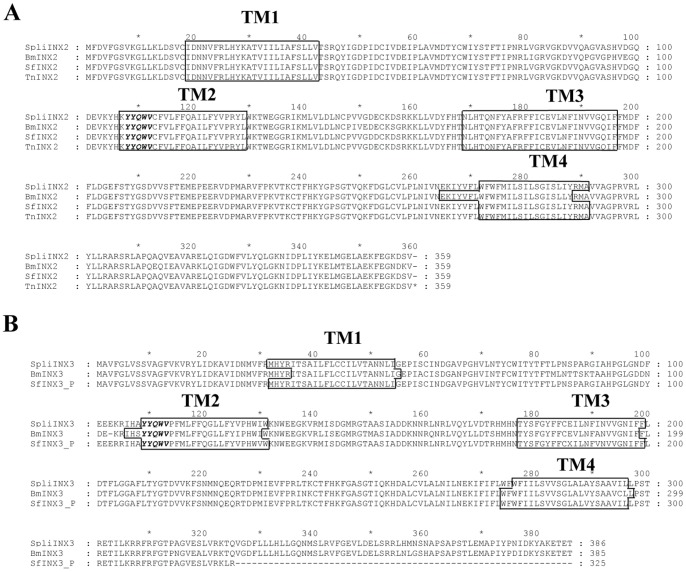
INX2 and INX3 from lepidopteran insects belong to a conserved innexin family, which contains a YYQWV motif at the beginning of the second TM domain. Alignment of the deduced amino acid sequence of SpliINX2 (A) with 3 other members of the Lepidoptera species, *S. frugiperda*, *B. mori* and *T. ni*, and SpliINX3 (B) with other 2 Lepidoptera species, *S. frugiperda*, *B. mori*. The predicted four transmembrane domains (TMs) are highlighted with frames, and the conserved motif YYQWV is highlighted in bold italic font. SpliINX2 (KC018471), Hi5INX2 (KC018473), BmINX2 (AAR97567), SfINX2 (AAP40732), BmINX3 (ACK38254), SpliINX3 (KC018472), and SfINX3_Partial (Sf1M11836-5-1, Sf2L00880-5-1 and Sf2L01032-5-1 from http://bioweb.ensam.inra.fr/spodobase/).

The *Spli-inx2* (KC018471) and *Spli-inx3* (KC018472) cDNAs were isolated from hemocytes of *S. litura* and are members of a conserved innexin family. The deduced amino acid sequences of SpliINX2 and SpliINX3 are shown in the figure 1. The published sequences of SfINX2 from *S. frugiperda* and BmINX2 and BmINX3 from *B. mori* were downloaded from GenBank. We also found three fragments, Sf1M11836-5-1, Sf2L00880-5-1, and Sf2L01032-5-1, using SPODOBASE that were predicted to belong to the INX3 family based on alignment results, and the consensus of these fragments was named SfINX3_Partial. Meanwhile, to understand more about the endogenous innexins in the insect cells, we also cloned the *Hi5-inx2* gene (KC018473). For this research, we obtained inx2 sequences from four Lepidoptera cells and inx3 sequences from three Lepidoptera cell lines. Interestingly, alignment of the deduced INX2 sequences showed high similarity, up to 96% (Figure 1A). Three of the transmembrane domains (TM1, TM2 and TM3) shared the same sequences. The TM4 domain of BmINX2 had only a few differences in the amino acid sequence. The alignment of INX3 showed almost identical results. The SfINX 3_partial also included the four transmembrane domains sequences (Figure 1B). A common motif, YYQWV, which had only been found in insect innexins [2], [8], was present at the beginning of the second TM of both INX2 and INX3. These results indicate that the INX2 and INX3 from four type of lepidopteran belong to the conserved innexin family and share highly similar sequences. Four Lepidoptera cells derived from different tissues could be used for testing whether the same innexins play different functions in different tissues. *Spli-inx2* and *Spli-inx3* genes were overexpressed in the endogenous cell line spli221 and ectopically expressed in Hi5 and Sf9 cell lines for functional testing.

Phelan [2] compared 61 innexin sequences and found that a pentapeptide, YY(X)W(Z), where X represents the amino acids Q, R, M, E or S and Z represents the amino acids V, M, A, I, S or T, is present close to the beginning of the second innexin TM domain in all invertebrate and insect-associated viruses; however, this motif, YYQWV, is highly conserved in insects. SpliINX2 and SpliINX3 present a common motif, YYQWV, at the beginning of the second TM domain. In addition to the four INX2s and three INX3s presented in this paper (Figure 1), recently sequenced *Spodoptera exigua* SeINX2 and SeINX3 also contain this conserved motif (personal communication with Dr. Fei Li). Recently, the transcription sequence of *S. litura* hemocytes has been finished in our lab; from these data, we also found *Spli-inx1* and *Spli-inx4* cDNA, which has YYQWV at the beginning of the second innexin TM domain (unpublished data). Although Yen et al., [8] indicate that innexins and pannexins from vertebrates belong to a single superfamily using statistical, topological and conserved sequence motif analysis, this insect innexin signature motif has not been found in the pannexin proteins. Connexin, pannexin, innexin and vinnexin have similar secondary structural folding, with four a-helices and intracellular N- and C- terminals. Through comparative studies of connexon, pannexon, innexon and vinnexon, it has been shown that hemichannels, from either invertebrates or vertebrates, may perform similar roles to regulate cell behavior.

### The Transient Expression by Transfection of Plasmids of Inx2 and Inx3 in Cells from Three Types of Lepidopteran Species

**Figure 2:**
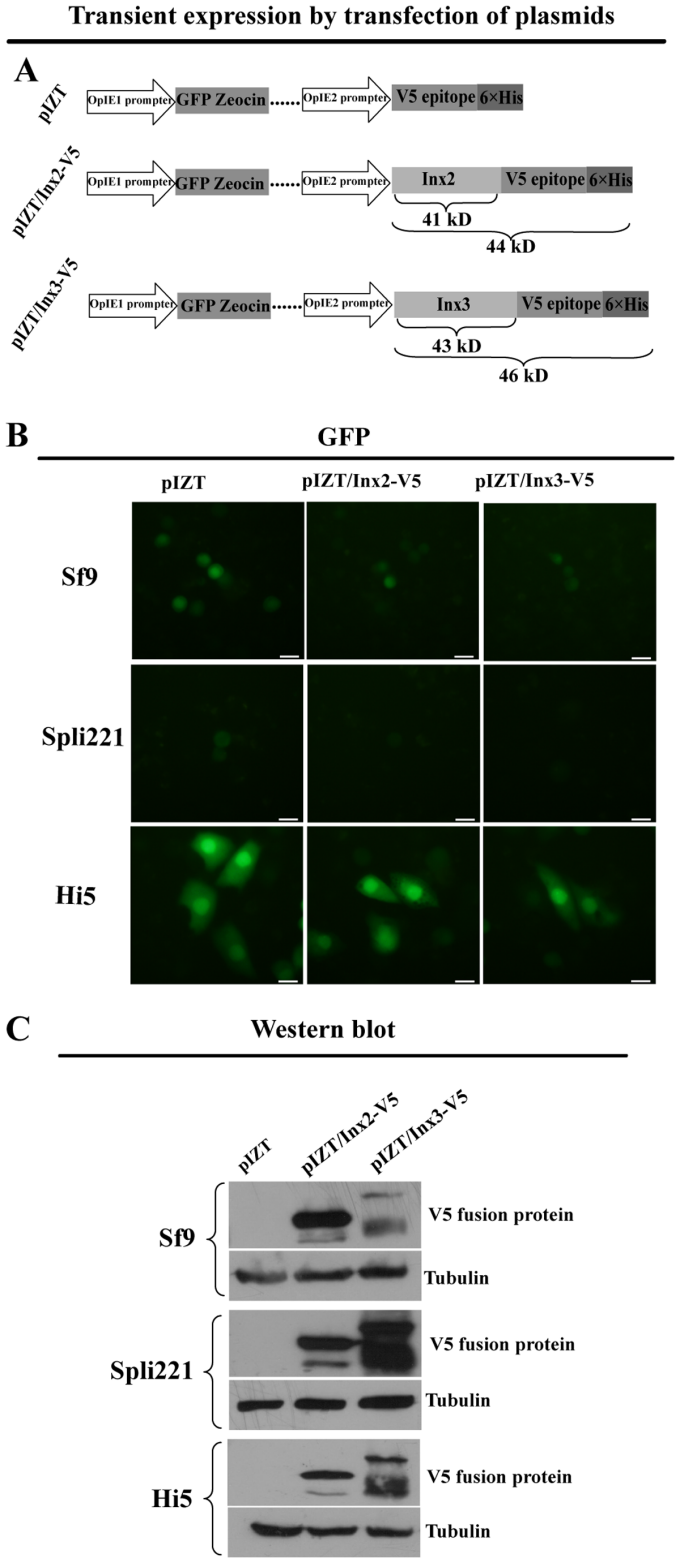
The transient expression by transfection of plasmids of Inx2 and Inx3 in cells from three types of Lepidopteran species. The construct of transfective plasmids (A), GFP was used to monitor transfection efficiency (B), and anti-V5 was used to visualize expression of the fusion proteins in cells (C). Empty plasmid vector (pIZT) was used as a negative control and tubulin was used as a loading control. Bar = 20 µm. (Hi5 derives from *T. ni*; Sf9 derives from *S. frugiperda*; Spli221 derives *S. litura*). Assays were repeated at least three times.

The Inx2-V5 fusion protein has a predicted molecular mass of 44 kDa, and the Inx3-V5 fusion protein has a predicted molecular mass of 46 kDa. GFP was used to identify the efficiency of transfection, Zeocin was used to select stable cell lines and a V5 antibody was used to identify the protein of interest (Figure 2A). Hi5 cells presented a high transfection efficiency, as determined by imaging at 96 h post-transfection (Figure 2B). Western blotting was used to identify proteins of interest. The same patterns were checked from three types of cells, and showed that exogenous innexin proteins were expressed in all cells tested (Figure 2C). These results led us to perform further experiments to determine whether these proteins correctly localized at the cell surface, indicating that innexins formed innexons. Based on the connexin life cycle, connexins oligomerize into hexameric connexons through the Golgi and then these connexons are transported to the plasma membrane [43], [44]. Six correctly folded domains form a hemichannel in the cell surface that is responsible for the exchange of ions and small molecules between the intracellular and extracellular space [45]. This communication plays crucial roles in activating the apoptotic signaling transduction pathway. For this purpose, we tried to establish stable cell lines with Zeocin selection.

### Ectopic Expression of Inx2 and Inx3 Promotes Apoptosis in Sf9 Cells

**Figure 3:**
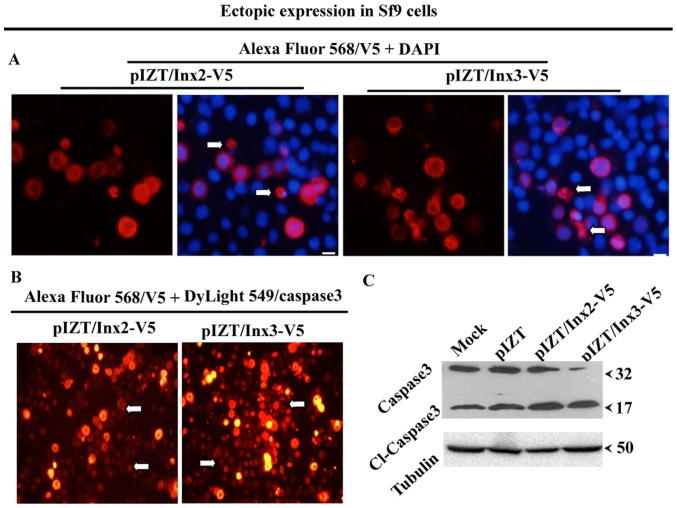
Ectopic expression Inx2 and Inx3 promote apoptosis in Sf9 cells. A. Inx2 and Inx3 localize on the cell surface, and apoptotic debris has been found (whit arrow showed). B. Inx2 and Inx3 proteins contributed to promote apoptosis of neighboring cells, as determined using an activated caspase 3-antibody (only label 17 kDa cleaved caspase 3) (Daylight 549-labeled secondary antibody shows rose color in the nuclei, white arrow) and V5-antibody (Alexa Fluor 568-labeled secondary antibody shows bright red color around membrane) observed via immunofluorescence microscopy. C. The expression level of cleaved caspase 3 was detected by western blot. Bar = 20 µm. Assays were repeated at least three times.

To create stable cell lines expressing Inx2 and Inx3, we first used the Sf9 cell line. However, using GFP as a transfection marker we observed that some cells expressing GFP appeared to be shrinking and looked as if they were undergoing apoptosis. Subsequently, we were unable to generate stable cell lines expressing either Inx2 or Inx3. Recent research reported that connexin 43 hemichannels promote apoptosis in vertebrate systems [22]. Thus, we sought to determine whether these shrinking cells were apoptotic and whether ectopic expression of Inx2 and Inx3 can promote apoptosis. First, we performed immunofluorescence assays to monitor apoptosis using the V5 antibody and DAPI. We observed that the V5 antibody labeled apoptotic bodies, suggesting that cells expressing inx2 and inx3 were undergoing apoptosis (Figure 3A). To further identify whether Inx2 and Inx3 transfection induced apoptotic signals in neighboring cells, two antibodies were used to investigate proteins of interest and apoptotic cells. The activated-caspase 3 antibody, which only labels cleaved 17 kDa caspase 3 (DyLight 549-labeled secondary antibody shows a rose color in the nucleus), labeled apoptotic cells, and the V5 antibody (Alexa Fluor 568 labeled secondary antibody shows bright red around membrane) labeled our protein of interest (Figure 3A). We found that the cells neighboring Inx2- and Inx3-expressing cells presented apoptotic signaling (white arrow shown)(Figure 3B). Western blotting was also used to monitor cleaved caspase 3, which can label the 32 kD pre-caspase 3 and the 17 kDa cleaved caspase 3, at 96 h post-transfection. The results showed that the 32 kDa pre-caspase 3 was cleaved to its functional 17 kD form. This result also showed that Sf9 cells underwent low level (<5%) apoptosis in normal cultural conditions; a 17 KD land was present (Figure 3C). These results suggest that Inx2 and Inx3 proteins can increase the percentage of death cells in cell types already showing a low level of cell death. These data are consistent with those from vertebrates that show the prominent function of hemichannels is to promote apoptosis: Connexons mediate apoptosis via the caspase cascade [14], [46], and the activation of the apoptotic caspase 3 generates irreversible cell death [47]. Connexin 43 colocalizes with caspase 3 in apoptotic hepatocytic cells, and connexin 43 inhibitors decrease the expression and activity of caspase 3 [20], [22]. Pannexon 1 find-me signals, and pannexon 1 itself is a target of caspase 3 [12], [14], [15].

### Over-expression of Inx2 and Inx3 Contributed to Apoptosis in Spli221 Cells

**Figure 4:**
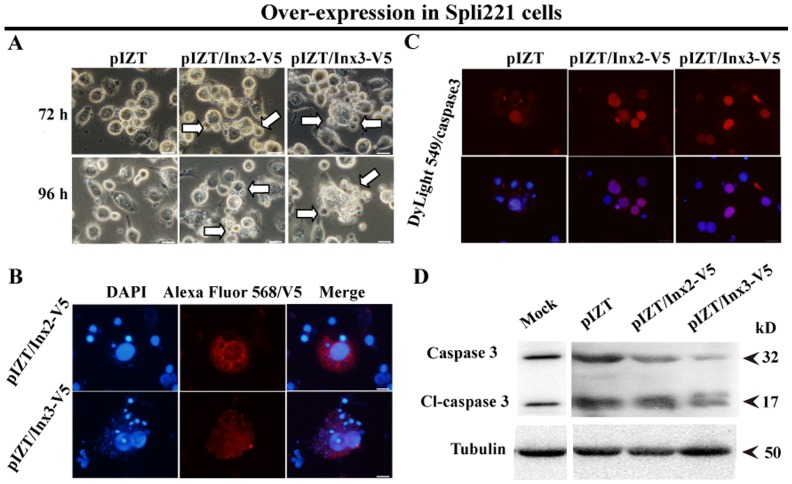
Over-expression of Inx2 and Inx3 contributed to membrane blebbing in Spli221 cells. A. Membrane blebbing was observed (white arrows) in Spli221 cells 72 and 96 hrs after transfection. B. Apoptotic bodies were observed by immunofluorescence in cells expressing Inx2 and Inx3. C. Activated caspase 3 localized to the nucleus of apoptotic cells, as determined by immunofluorescence. D. Levels of cleaved caspase 3 were detected by western blot. Bar = 20 µm. Assays were repeated at least three times.

We were unable to establish stable protein expression in the homologous cell line, Spli221. Interestingly, large membrane blebbing was observed in those cells that over-expressed Inx2 and Inx3 (Figure 4A, white arrow shown). To confirm that this membrane blebbing was due to apoptosis, we used immunofluorescence and western blotting to monitor apoptosis. Apoptotic bodies were observed in cells expressing Inx2 and Inx3. Small nuclei, stained by DAPI were present around the cells expressing Inx2 and Inx3 (Figure 4B). Activated caspase 3 was used to detect apoptotic cells. A DayLight 549-labeled secondary antibody stained apoptotic nuclei, which were also co-stained with DAPI (Figure 4C). Western blotting showed that the apoptotic ratio in cells expressing Inx3 was larger than that of cells over-expressing Inx2. Like Sf9 cells, Spli221 cells underwent low-level apoptosis in normal culture conditions, as a 17 kD band is also seen in mock transfected cells (Figure 4D). These results indicate that over expression of Inx2 and Inx3 promotes apoptosis.

Our data show that insect innexons promote apoptosis in Sf9 and Spli221 cell lines and promote membrane blebbing during apoptosis in the Spli221 cell line. In vertebrates, the occurrence of apoptotic membrane blebbing is dependent on the function of ROCK I, which is cleaved during apoptosis by activated caspases [48] and the activation of caspase 3 is concomitant with the inactivation of the PI3K/Akt signaling pathway during apoptosis [26].

### Inx2 and Inx3 Localize to the Cell Surface through Stable Expression in Hi5 Cell Lines

**Figure 5:**
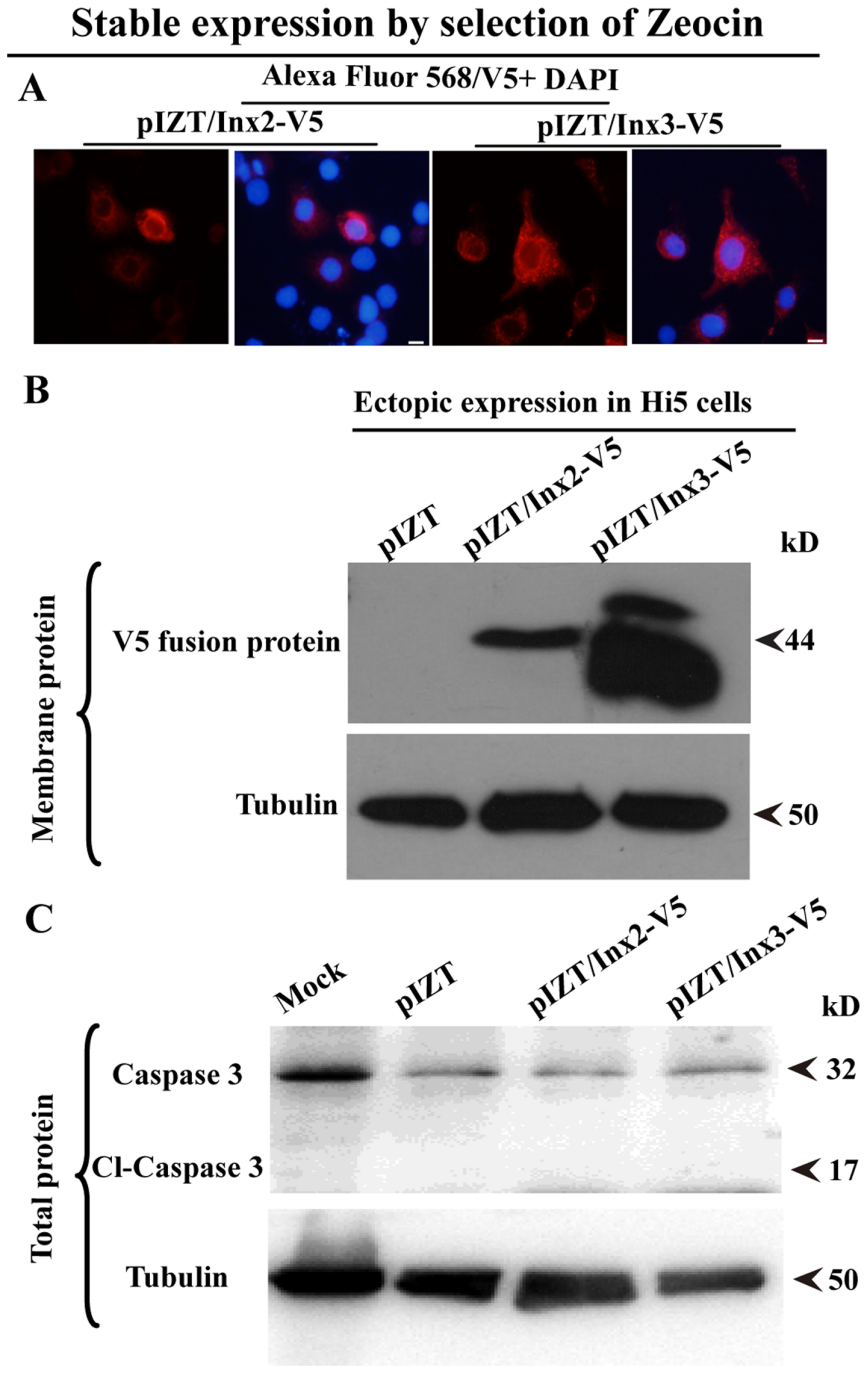
Inx2 and Inx3 localized in the cell surface of stable expression in Hi5 cells. A. Immunofluorescence identify the localization of protein of interesting. B. The surface innexons was isolated by biotinylation. C. Absence of caspase cleavage was identified using a caspase 3 antibody. Assays were repeated at least three times.

Although we were unable to generate stable cell lines expressing Inx2 or Inx3 in Sf9 and Spli221 cell lines, through Zeocin selection we succeeded in generating stable clone expressing Inx2 and Inx3 in the Lepidoptera Hi5 cell line. In these Inx2 and Inx3 stable cell lines, we observed that Inx2 and inx3 localized to the cell surface (Figure 5A). Additionally, using surface protein isolation biotinylation assays, we confirmed that innexons were localized in the cell membrane (Fig. 5B). The levels of Inx3 in the cell membrane were higher than those of Inx2; inx3 protein migrates as two bands, which might be characteristic of post-translational modification, degradation or a ribosomal reading issue (there is an AUG codon approximately 90 nt into the *Spli-inx3*) (Figure 5B).

Insect innexons localized in the cell membrane have the ability to perform a similar function. In insect immune suppression, *C. sonorensis* ichnovirus vinnexin plays an extraordinarily prominent role in disrupting the cellular immune response. Vinnexin Q2 localizes in the cell surface of hemocytes infected by CsIV [5]. Ectopic expression of vinnexon Q1 and vinnexon Q2 in the Hi5 cell line showed distribution of these proteins in the cell membrane, and the vinnexons also localized to the cell cytoplasm (personal communication with Dr. Turnbull) and docked together, forming gap junctions [9]. Innexins and Vinnexins ectopically expressed in the Hi5 cell line showed the same results: localization to the cell surface membrane, and the establishment of stable cell lines.

Expressed innexins in lepidopteran cell lines were distributed predominantly in the cytoplasm and membrane, but also could also be found localized in the nucleus. Connexon [22], [43], [44] and pannexon [14], [21] proteins also localize in the cell surface. One post-transcriptional modification in connexins is phosphorylation [20], [44], and mutation of connexins leads to a lack of phosphorylation [46].

To understand why we were successful in generating stable expressing cell lines for either Inx2 or Inx3 in Hi5 cells when it was not possible in Sf9 and Spli221 cells, we investigated the status of apoptosis in Hi5 cells. In the pIZT, pIZT/Inx2-V5 and pIZT/Inx3-V5 cell lines, no cell death was observed by microscopy (Figure 3A). Furthermore, through western analyses using a caspase 3 antibody, a 32 kDa pre-caspase 3 band was observed in the pIZT, pIZT/Inx2-V5 and pIZT/Inx3-V5 cell lines, while no 17 kDa cleaved caspase 3 band was visible (Figure 5C). Collectively, these data show that following ectopic expression of inx2 and inx3 in Hi5 cells, innexons localized in the cell membrane without altering cell behaviors nor promoting apoptosis.

### Apoptosis Promoted by Innexon Expression in Lepidoptera Cells May Involve the Inactivation of the PI3K/Akt Signaling Pathway

**Figure 6:**
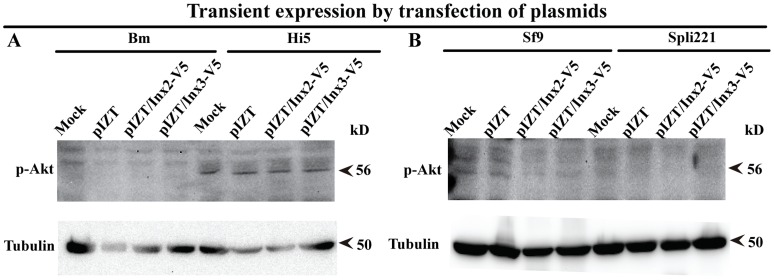
Apoptosis promoted by innexons may involve the inactivation of PI3K/Akt signaling pathway. P-Akt has been detected by using p-Akt antibody, Bm12 cell as a control of Hi5 cells (A) and the inactivation of PI3K/Akt signaling pathway presented in apoptosis cells in Sf9 and spli221 (B). Assays were repeated at least three times.

Why the expression of Inx2 or Inx3 in Sf9 or Spli221 cells promoted apoptosis, whereas expression in Hi5 cells did not, raises the question of how the same protein affects cell behavior differently in different cell types? Thus, since apoptosis involves the inactivation of the PI3K/Akt signaling pathway and the activation of caspase cascades [26], [27], we wished to assay whether the expression of innexons affected the PI3k/Akt signaling pathway: In Sf9 and Spli221 cells, which undergo apoptosis upon the expression of Inx2 and Inx3, the PI3K/Akt signaling pathway should be inactivated, whereas in Hi5 cells, where there is no apoptosis even upon Inx2 and Inx3 expression, the PI3K/Akt signaling pathway should be activated. We observed that Sf9 and Spli221 cells showed inactivated PI3K/Akt signaling pathway, whereas in Hi5 cells in either Inx2, Inx3 or mock-transfected cells, the PI3K/Akt signaling pathway was activated (Figure 6). Thus, given that in vertebrates, the PI3K/Akt signaling pathway regulates downstream pathways associated with cell survival and baculoviruses are well know to inhibit apoptosis by activating PI3K/Akt signaling pathways to maintain cell survival for replication of virus [31], [35], it may be that Inx2- and Inx3-induced apoptosis in Sf9 and Spi221 cells is due to inactivated PI3K/Akt signaling, although further studies are required to determine the exact pathways involved.

### Conclusions

Hemichannels are powerful structures for communication in the cell membrane that serve to regulate cell behaviors via the bidirectional passage of small molecules between the intracellular and extracellular space, leading to activation of paracrine signaling pathways [16]. Previous studies have shown that insect hemichannels are involved in insect cellular immune responses [9], [28], but the molecular mechanisms still remain unknown. In this study, we report that Inx2 and Inx3 play an important role in promoting apoptosis in insect cell types already showing a low level of cell death. The apoptogenic properties of innexons were demonstrated using the *Spli-inx2* and *Spli-inx3* cDNA, isolated from *S. litura* hemocytes. The cultured cell lines, Sf9 and Spli221 normally underwent low levels of apoptosis and this was markedly increased through the ectopic expression of innexons. Together, these data suggest that insect innexons play a role in promoting apoptosis. Further data also suggest that the inactivation of PI3K/Akt signaling pathway may be involved in cell apoptosis. These findings will facilitate further examinations of the apoptotic regulation by the PI3K/Akt signaling pathway and comparative studies of innexons, connexons, pannexons, and vinnexons.
